# Supplementary material for: Diet-dependent natriuretic peptide receptor C expression in adipose tissue is mediated by PPARγ via long-range distal enhancers
Source: J Biol Chem. 2021 Jul 7;297(2):100941. doi: 10.1016/j.jbc.2021.100941 (PMC8326739; doi:10.1016/j.jbc.2021.100941)
Supplement: Supporting Table [file mmc1.docx]

**Supporting Information: Primer sequences**

| **QPCR** | **Fwd (5’-3’)** | **Rev (5’-3’)** |
| --- | --- | --- |
| 36B4 | GATGCCCAGGGAAGACAG | ACAATGAAGCATTTTGGATAATCA |
| Nprc | AGCTGGCTACAGCAAGAAGG | CGGCGATACCTTCAAATGTC |
| Pparg2 | tgctgttatgggtgaaactctg | ctgtgtcaaccatggtaatttctt |
| Pparg | gaaagacaacggacaaatcacc | gggggtgatatgtttgaacttg |
| Fabp4 | ggatggaaagtcgaccacaa | tggaagtcacgcctttcata |
| Npra | TGGAGACACAGTCAACACAGC | CGAAGACAAGTGGATCCTGAG |
| eNprc-49k | GAACAATGCCTGACACATGG | CTTTGTGGCAAGCTCCTCTC |
| eFabp4-5.4k | TGTGCATTTCTGCCTTTGTC | GAATTCCCAGCAGGAATCAG |
| **3C PCR** | **Enhancer Bait (5’-3’)** | **Promoter Anchor (5’-3’)** |
| Nprc-9k | GTCTGTATTTCTGAGGAGTGCTGTA | AGCCTTCCTTTTAGATTCCTTCCAT |
| Nprc-44/49k | GTTGTTTCCCTGAATTCCTAAACCC | AGCCTTCCTTTTAGATTCCTTCCAT |
| Nprc-51k | CTTCTCATGGCCTTTGTGTGTTAAT | AGCCTTCCTTTTAGATTCCTTCCAT |
| Nprc-54k | TACTGTTCAAGCCATCACAGAACTA | AGCCTTCCTTTTAGATTCCTTCCAT |
| Nprc-58k | TCCTGTAGCTGTCCAGTCCA | AGCCTTCCTTTTAGATTCCTTCCAT |
| Nprc-62k | TGCTAACTGGATGCCACTGG | AGCCTTCCTTTTAGATTCCTTCCAT |
| Nprc-71k | CTCATGAAGGAGGGACAGGAGGAGCTG | AGCCTTCCTTTTAGATTCCTTCCAT |
| Ercc3 | CACATGCCCTCCCTGAAAATAAG | CTAAAGAAGAGCAGGAGGTTTCAGA |
| β-actin | CTGTCGAGTCGCGTCCACCC | CCGCACCGGCTCATCAAATG |
| **Reporters** | **Fwd (5’-3’)** | **Rev (5’-3’)** |
| Nprc-2.2k | ATGACTCGAGCCGGCACCCGGTATGAAGTT | ATTAAAGCTTCTCGCCCTCTGCCCCCCAC |
| Nprc-9k | ATGCGTCGACTGGAGGAGGCTTTGAGATCC | ATGCGGATCCGTCCCACACCAAGACGTAGA |
| Nprc-44k | ATGCAAGCTTAGCTGCAGGATCTCTACGAC | ATGCGTCGACGGTAGAGGCAGGTGGATCTC |
| Nprc-49k | ATGCGTCGACAGCAAAGCATACTTGCTGGGAAG | ATGCGGATCCAAGAGAGCTGGGACCTTCAGGAC |
| Nprc-51k | ATTGTTGTCGACGCGCCAGGAAGTAAGTTGAG | ATTGTTGGATCCGCTGAACATGCTGGTTTCCA |
| Nprc-54k | ATGCGTCGACCTCAATGAACTTCTATTTAGTTTGTATCAC | ATGCGGATCCTGGTCATTATTTTCCACTTGAGCAGC |
| Nprc-58k | ATGCGTCGACTGTCTCCCTCCCTCCATGTA | ATGCGGATCCCTTGGTTTTCACTTTCAGTTCAGT |
| Nprc-62k | ATGCGTCGACAGGAATGGACAGAAAG | ATGCGGATCCATTTTGAAGTCGGGCTGGTG |
| Nprc-49k-P1 | ATTGTTGTCGACAGTTTGCAAGAAGAAAGACAGCT | ATTGTTGGATCCACTCCTGAAAGTTGTCCTCTGAT |
| Nprc-49k-P2 | ATTGTTGTCGACATACAAACAGGAACCTCAACTCC | ATTGTTGGATCCCCCAAGTCAGCAGCTAATCATAG |
| Nprc-49k-P3 | ATTGTTGTCGACGTTGAGGGAAGGACATTGATCTG | ATTGTTGGATCCACACCACAAGAGATAGCCAGATA |
| **PPRE Oligos** | **Fwd (5’-3’)** | **Rev (5’-3’)** |
| PPRE-49k-P2 | TCGACAATAAAGTAGGTCAAAGACCAGAGAGTCATG | TCGACATGACTCTCTGGTCTTTGACCTACTTTATTG |
| PPRE-49k-P2M | TCGACAATAAAGTAAATGAAAAACGAGAGAGTCATG | TCGACATGACTCTCTCGTTTTTCATTTACTTTATTG |
